# Determination of the Cytotoxic Effect of Different Leaf Extracts from *Parinari curatellifolia* (Chrysobalanaceae)

**DOI:** 10.1155/2020/8831545

**Published:** 2020-10-28

**Authors:** Anesu Kundishora, Simbarashe Sithole, Stanley Mukanganyama

**Affiliations:** ^1^School of Pharmacy, College of Health Sciences, University of Zimbabwe, Mt. Pleasant, Harare, Zimbabwe; ^2^Department of Biochemistry, University of Zimbabwe, Mt. Pleasant, Harare, Zimbabwe

## Abstract

Despite plants being a rich source of useful chemical compounds with different pharmacological properties, some of these compounds may be toxic to humans. *Parinari curatellifolia*, among its other important pharmacological activities, has been shown to have significant antiproliferative activity on cancer cell lines. Toxicity studies are required to determine the safety profile of *P. curatellifolia* in the consideration of its potential pharmaceutical benefits as a source of lead compounds in cancer therapy. The effects of *P. curatellifolia* on both the integrity of the erythrocyte membrane and on normal cells were determined. The dried leaf powder of *P. curatellifolia* was used in serial exhaustive extraction procedures using hexane, dichloromethane, ethyl acetate, acetone, ethanol, methanol, and water as solvents in addition to extraction using DCM: methanol in equal ratio. Alkaloids, flavonoids, and saponins were isolated from the ethanol extract. The leaf extracts were tested for haemolytic activity on sheep erythrocytes at concentrations of 0.625 to 5 mg/ml. The extracts were also tested for toxicity activity on normal mammalian cells such as the BALB/c mice peritoneal cells using the MTT (3-(4,5-dimethylthiazol-2-yl)-2,5-diphenyltetrazolium bromide) at the concentrations of 6.3 to 50 *μ*g/ml. In the haemolysis assays, none of the plant extracts had a significant haemolytic activity with the saponin-enriched extract having the maximum haemolytic activity of 12.2% for a concentration of 5 mg/ml. In the MTT cell viability assay, none of the 11 plant extracts had significant cytotoxicity. The water extract, however, had significant (*p* < 0.01) proliferative activity towards the murine immune cells at all concentrations*. P. curatellifolia* leaf extracts were, therefore, not toxic to both erythrocytes and immune cells, and the water extract may have immunostimulatory effects. It is concluded that *P. curatellifolia* leaf extracts are not toxic *in vitro* and, therefore, our results support the use of the plant for ethnomedicinal use.

## 1. Introduction

Herbal medicines have been the heart of treatment and cure for various ailments and physiological conditions in ethnomedicinal practices [1, 2]. Eighty percent of African populations use some form of traditional herbal medicine, and the worldwide annual market for herbal products approaches US$ 60 billion [3]. Chemical compounds from plants such as flavonoids, terpenes, alkaloids, anthraquinones, saponins, tannins, steroids, lactones, and volatile oils have received considerable attention in recent years due to their varied pharmacological properties, including cytotoxic and chemopreventive effects [4]. About 80% of the population in developing countries use traditional medicines because they cannot afford the high cost of western pharmaceuticals and health care as well as that traditional medicines are more acceptable from a cultural and spiritual perspective [5]. In Zimbabwe, it is estimated that out of more than 5000 plant species, about 10% are used in ethnomedicine [6].

*Parinari curatellifolia* is one of the plants whose parts have been widely used for therapeutic purposes both locally and continentally. *P. curatellifolia* is an important plant in traditional medicine in sub-Saharan Africa. The plant is used in traditional medical practices in the treatment of cancer, pneumonia, fever, malaria, typhoid, hypertension, microbial infections, pain, anti-inflammation, toothaches [6], dressing of fracture, and dislocations [7]. Three cytotoxic diterpenoids were isolated from *P. curatellifolia* root bark [8], two being novel compounds, namely, 13-methoxy-15-oxozoapatlin and 13-hydroxy-15-oxozoapatlin, and 15-oxozoapatlin.

Despite plants being a rich source of beneficial chemical molecules of diverse structures with different pharmacological properties on biological systems [9, 10], some plants may be toxic to humans. For example, some of the toxicities related to the use of medicinal plants include allergic reactions, irritation of the gastrointestinal tract, destruction of red blood cells, and injury to vital body organs such as the heart, liver, kidney, and carcinogenicity [11]. Most important causes of medicinal herb toxicity are the presence of toxic phytochemicals in the medicinal plant, improper identification or verification of herbals, and inappropriate or mislabelling of the plant material. Other causes are contamination of herbals with microorganisms, fungal toxins such as aflatoxins, and interactions with standard drugs upon simultaneous ingestion [12–14].

A majority of toxicological studies indicate that the toxic effects in herbal medicines are associated with hepatotoxicity [15]. Other harmful effects on the nervous system, kidneys, blood and cardiovascular system, as well as mutagenicity and carcinogenicity, have been reported [15, 16]. Studies to assess the potentially harmful effects of herbal medicines and other chemical compounds can be carried out both *in vitro* and *in vivo* [17–19].

The peritoneal cavity is a membrane-bound and fluid-filled mammalian abdominal cavity, which houses the liver, spleen, greater percentage of the gastrointestinal tract, and other viscera [20]. The peritoneal cavity provides an uncommon setting for immune interactions as it is geared up to respond rapidly to bacteria that may be released by intestinal spillage [21].

Toxicity studies were carried out to address the safety of the *P. curatellifolia* leaf extracts on the integrity of the erythrocyte membrane as well as on normal somatic cells such as immune cells. The study will serve as a toxicology reference for further investigations on therapeutic practises and drug development research studies involving the use of *P. curatellifolia.* This study was aimed at investigating and determining the cytotoxic effect of different *P. curatellifolia* leaf extracts on normal mammalian cells.

## 2. Materials and Methods

### 2.1. Chemicals and Reagents

All the chemicals used in this study were obtained from Sigma-Aldrich Chemicals Company (Munich, Germany). Acetone, dichloromethane, ethanol, ethyl acetate, and hexane were used as the extraction solvents. Dimethyl sulfoxide was used for dissolving extracts. Drabkin's reagent was used in the haemoglobin content determination assay. Daunorubicin was used as the standard cytotoxic drug, and MTT was used as an indicator of cell viability. Roswell Park Memorial Institute (RPMI) medium was used for growing the mammalian cells *in vitro*. A Celestron digital light microscope (Celestron Electronics, California, USA) and a haemocytometer were used in the determination of peritoneal cell count. All incubations were done in a Shel Lab incubator (Sheldon Manufacturing, Inc., Cornelius, USA), and for analysis of cell density, a GeniosPro microplate reader (Tecan Group Ltd, Mӓnnedorf, Switzerland) was used.

### 2.2. Ethical Consideration

The study was approved by the Joint Parirenyatwa Hospital and College of Health Sciences Research Ethics Committee (JREC 334/16, Harare, Zimbabwe), and permission to use mice was granted by the Animal House Department, under the Faculty of Veterinary Science. Animals were maintained and handled according to the recommendations of the good laboratory practice and animal handling (NIH) guidance for the care and use of laboratory animals, publication no. 85-23, 1985.

### 2.3. Plant Collection

*P. curatellifolia* fresh leaves were collected from Centenary (latitude: 16°48′00″S, longitude: 31°07′00″E and elevation above sea level is 1156 m) in the Mashonaland Central Province of Zimbabwe. Mr. Christopher Chapano, a taxonomist at the National Herbarium and Botanical Gardens (Harare, Zimbabwe), authenticated the identity of the plant. A voucher reference specimen (C6E7) was kept at the Biomolecular Interactions Analyses (BIA) Laboratory, in the Department of Biochemistry, at the University of Zimbabwe. The leaves were air-dried and ground using a pestle and mortar. The resulting leaf powder was stored in closed, labelled containers at room temperature.

### 2.4. Preparation of Plant Extracts

*P. curatellifolia* powdered leaves were used in total extraction process, using dichloromethane: methanol as cosolvents in equal ratio (50 : 50). Serial exhaustive extraction included the use of seven solvents of different polarities to obtain seven extracts. The sample to solvent ratio was 1 : 5. The solvents used for serial exhaustive extraction were beginning with hexane, dichloromethane, ethyl acetate, acetone, ethanol, methanol, and ending with water. Extraction was carried out for 24 h on a magnetic stirrer at moderate speed. After extraction, the mixture was filtered first with cotton wool followed by the use of Whatman No. 1 filter paper (Sigma-Aldrich Chemicals, Steinheim, Germany), and the filtrate was left to dry in centrifuge tubes. The residue from one stage of serial exhaustive extraction was dried and resuspended in the next solvent, and extraction procedure was repeated until the last solvent has been used. The dried extracts were physically examined, labelled, and stored at 4 C.

### 2.5. Isolation of Specific Phytochemicals

The alkaloids, flavonoids, and saponins were isolated from *P. curatellifolia* leaves according to the methods by Aslantürk [22]. From the crude ethanolic extract powder, an equal mass of the extract was put into three different labelled tubes. In tube 1, the powder was mixed with ammonia solution and extracted with 80% methanol for 10 minutes in a water bath at 40°C, to extract alkaloids. The powder in tube 2 was heated with 80% methanol on a water bath at 40°C for 10 min to isolate flavonoids. The extracts were filtered through Whatman No. 1 (Sigma-Aldrich Chemicals, Steinheim, Germany) and the filtrates were concentrated by air-drying under a fan and stored at 4°C. For saponins isolation, the plant sample was mixed with 20% aqueous ethanol and heated in a water bath for 90 min at 55°C. The mixture was filtered first with cotton wool followed by the use of Whatman No. 1 filter paper (Sigma-Aldrich Chemicals, Steinheim, Germany), and the residue was re-extracted with 20% ethanol. Filtrates were mixed. An equal volume of diethyl acetate was added, and re-extraction was carried out by partitioning until the aqueous layer became clear. Saponins were extracted using *n*-butanol and washed using 5% aqueous sodium chloride. The resulting saponins sample was fan-dried, physically analysed, and stored at 4°C.

### 2.6. Haemolysis Assay

Sodium citrate was added to a flask in which sheep blood that was aseptically collected from the Animal House at the University of Zimbabwe was kept. An equal volume of Alsever's solution (Sigma-Aldrich), pH 7.4, an anticoagulant, was added. The blood was centrifuged at 3 000 rpm for 10 min, and the supernatant was discarded. The residue was washed with a 1 : 5 volume of phosphate-buffered saline (PBS), pH 7.4, by centrifuging at 4 000 rpm for 5 min. The supernatant was discarded, and the cells were diluted fourfold with PBS to make a 25% sheep erythrocyte suspension. The resulting suspension was used for the determination of haemolysis. The erythrocyte suspension (500 *µ*l) was incubated with an equal volume of test sample extracts dissolved in PBS, in varying concentrations from 0.625 to 5 mg/ml, in 1.5 ml microtubes (Eppendorf, USA) for 90 min at 37°C. After incubation, the tubes (including the negative control tube) were spun in a microcentrifuge at 3000 rpm. A volume of 3 ml of Drabkin's reagent was added to 200 *µ*l of resulting supernatant. The positive control contained uncentrifuged erythrocyte suspension and buffer, while the negative control contained centrifuged erythrocyte suspension and buffer. Aliquots of the supernatants in Drabkin's reagent were added to 96-well plates. To determine the amount of haemoglobin released, the absorbance of samples was determined at 590 nm in a GeniosPro microplate reader (Tecan Group Ltd, Mӓnnedorf, Switzerland). The percentage haemolysis was calculated as follows [23]:

(1) $$\begin{array}{c} \frac{\left(\text{simple absorbance }-\text{negative control absorbance}\right)}{\left(\text{positive control absorbance }-\text{negative control absorbance}\right)}\times 100\text{\%.} \end{array}$$

### 2.7. Toxicity Testing on Murine Peritoneal Cells

#### 2.7.1. Induction and Harvesting of Immune Cells

Male BALB/c mice of 8 weeks old were obtained from the Animal House at the University of Zimbabwe (Harare, Zimbabwe). The intraperitoneal route was used to inject 6 mice with a volume of 1 ml of 20% starch solution. The mice were left for 24 h to increase the number of cells in the peritoneal cavity. Each mouse was euthanised by cervical dislocation. The mouse was sprayed with 70% ethanol and mounted on a styrofoam board on its back. The outer skin was cut to expose the inner skin in the peritoneal cavity. A volume of 5 ml of cold PBS containing 3% FBS was injected into the peritoneal cavity taking care not to puncture any organs. Cells were withdrawn using a 10 ml syringe, and collected cells were put into tubes and kept on ice. The collected cell suspension was centrifuged, the supernatant was discarded, and the cells were resuspended in RPMI. Cells containing blood were thrown away as they had been contaminated. Cells of a volume of 100 *µ*l were mixed with an equal volume of 0.4% trypan blue staining dye and counted manually using a haemocytometer counting chamber under a Celestron digital light microscope (Celestron, Los Angeles, USA) to determine the number of viable cells. The cells were incubated for overnight at 37°C and at 5% CO_2_ in a Shel Lab incubator (Sheldon Manufacturing, Inc., Cornelius, USA).

#### 2.7.2. MTT Assay

Murine cells of a volume of 100 *µ*l were mixed with an equal volume of 0.4% trypan blue staining dye and counted manually using a haemocytometer counting chamber under a Celestron digital light microscope to determine the number of viable cells. The cells were plated onto 96-well plates and incubated with the extracts of concentrations 6.3, 12.5, 25, and 50 *µ*g/ml for 48 h at 37°C in a 5% CO_2_ incubator. The negative control contained cells and RPMI complete media only, and positive control contained cells, RPMI complete media, and daunorubicin, an anticancer drug. RPMI complete medium (in a 500 ml bottle) was made by mixing the following: 89% RPMI medium, 10% foetal bovine serum (FBS), and 1% PNS, a cocktail of antibiotics comprising penicillin, neomycin, and streptomycin. After the 48 h incubation, 25 *µ*l of MTT was added to each well and plates were incubated for 4 hours. A volume of 50 *µ*l of dimethyl sulfoxide (DMSO) was added and absorbance was measured at 590 nm using a Tecan Genios-Pro microplate reader (Tecan Group Ltd, Mӓnnedorf, Switzerland).

### 2.8. Statistical Analyses

Graphical and statistical analyses will be carried out using GraphPad Prism Version 6. All data were expressed as mean ± standard deviation of the mean. One-way analysis of variance test (ANOVA) with Dunnett's multiple comparison post hoc test was used to analyse the results. Difference in *p*-values of 0.05 or less was considered significant.

## 3. Results

### 3.1. Crude and Serial Exhaustive Extraction

**Figure 1:**
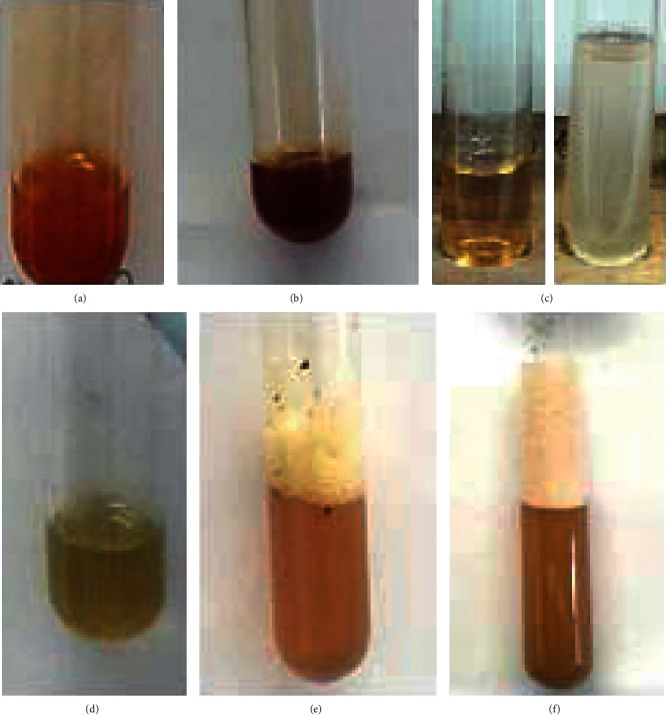
Qualitative analysis results. Tests for alkaloids: (a) Dragendorff's reagent test and (b) Wagner's reagent test. Tests for flavonoids: (c) sodium hydroxide test, (d) ferric chloride test, and (e) Shinoda test. Test for saponins: (f) frothing test.

Serial exhaustive extraction had a higher percentage yield than crude extraction. Crude extraction had a percentage yield of 5.51%, and serial exhaustive extraction had a total percentage yield of 18.81%. The methanol extract had the highest percentage yield, with the hexane extract having the least yield. Three fractions of phytochemicals were isolated from crude ethanolic extract of *P. curatellifolia* leaves. The fractions' colour, consistency after drying, and percentage yield are shown in Table 1 and Figure 1.

### 3.2. Haemolysis Assays

**Figure 2:**
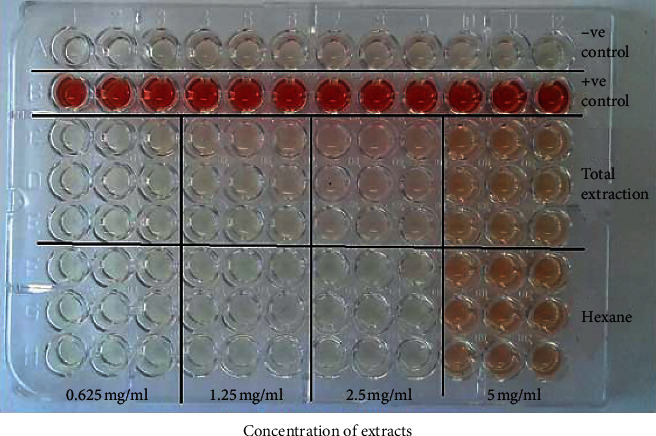
A 96-well plate for haemolysis of sheep erythrocytes exposed to *P. curatellifolia* crude (total) and hexane extract. The negative control (−ve control) was a measure of spontaneous haemolysis and contained centrifuged erythrocyte suspension in PBS from which an aliquot of the supernatant was withdrawn and mixed with Drabkin's reagent. The positive control (+ve control) contained uncentrifuged erythrocytes in PBS from which an aliquot was withdrawn and mixed with Drabkin's reagent to give 100% haemolysis.

**Figure 3:**
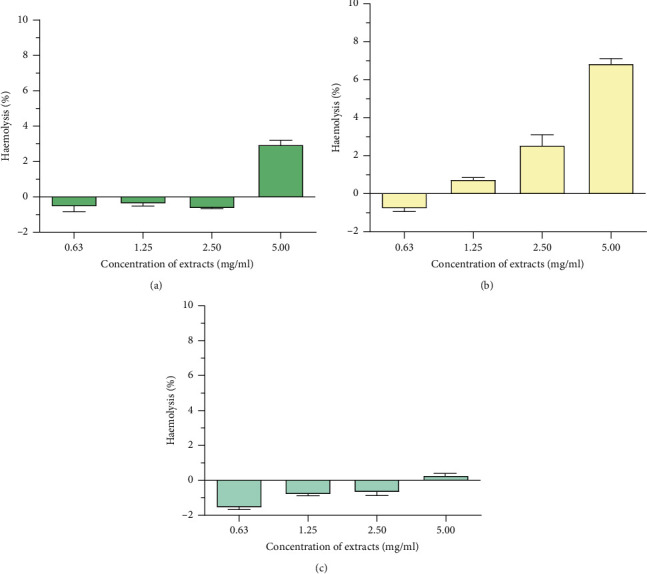
The effect of extracts of *P. curatellifolia* on haemolysis of sheep erythrocytes. (a) DCM: methanol extract, (b) water extract, and (c) hexane extract. The erythrocyte suspension was incubated with an equal volume of test sample extracts; A, B, and C dissolved in PBS, at varying concentrations from 0.625 to 5 mg/ml at 37°C.

**Figure 4:**
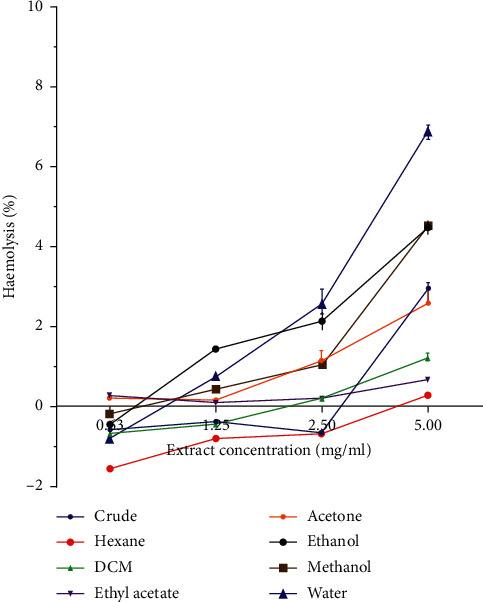
Comparison of the haemolytic activity of all serial exhaustive leaf extracts with respect to the crude extract. The water extract had the highest haemolytic activity. Crude is the DCM: methanol extract.

**Figure 5:**
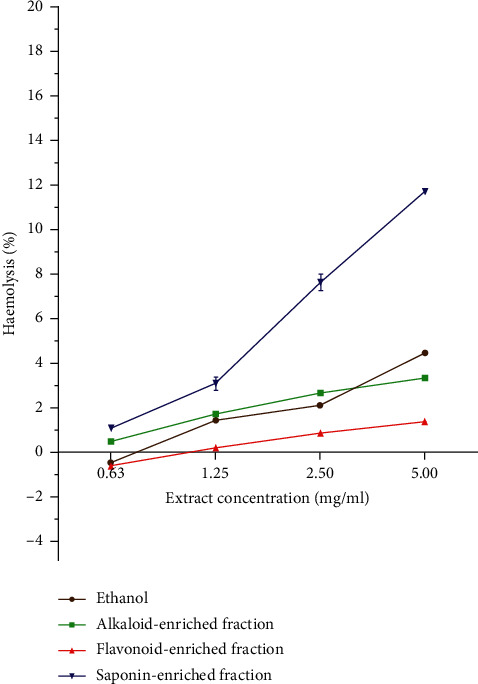
Comparison of the haemolytic activity of phytochemical-enriched fractions with respect to the ethanolic extract from which they were obtained. The saponin-enriched fraction exhibited the highest activity.

Haemolytic activity of *P. curatellifolia* leaf extracts was assessed against sheep erythrocytes to determine the effect of the plant on erythrocyte membrane integrity (Figure 2). The activity of the plant extracts was expressed in percentage haemolysis and reported as mean ± standard deviation of three replicates. Plant extracts are considered toxic to red blood cells if the percentage haemolysis is above 30% [24]. All extracts exhibited low haemolytic activity as activity in all extracts was less than 10% (Figures 3 and 4). The saponin-enriched fraction had the highest percentage haemolysis of 12.2% (Figure 5). All extracts showed a dose-dependent increase in haemolytic activity.

### 3.3. In Vitro Cytotoxicity Assay Using Mice Peritoneal Cells

**Figure 6:**
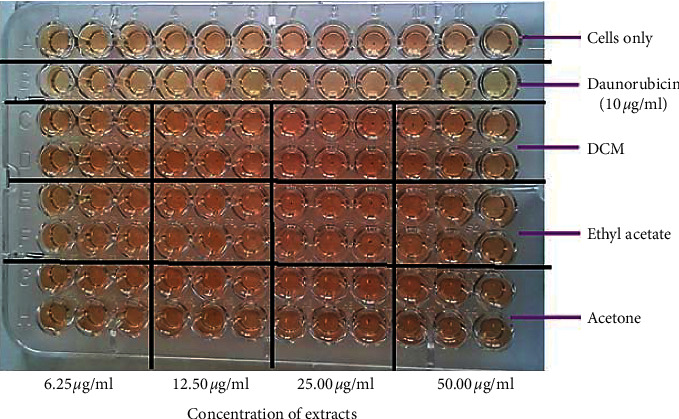
Image showing a 96-well plate for the MTT assay using mouse peritoneal cells, exposed to different solvent extracts from *P. curatellifolia* leaves (black dots in the wells indicate purple-coloured peritoneal cells that have sedimented at the bottom of the well). Cells only in row were the negative control, while the positive control was daunorubicin 10 *μ*g/ml, a standard anticancer drug.

**Figure 7:**
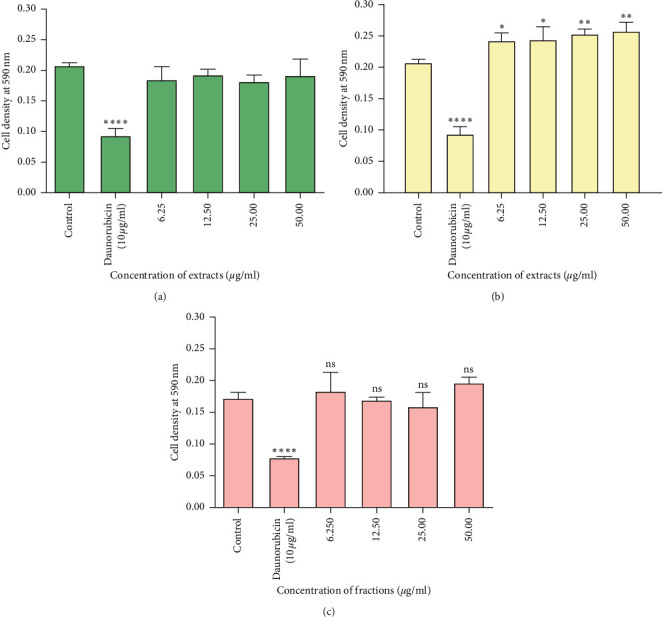
The effects of the leaf extract from *P. curatellifolia* on the viability of mouse peritoneal cells using the MTT assay. (a) Methanol extract, (b) water extract, and (c) saponin-enriched fraction. Mice peritoneal cells were plated in 96-well plates and incubated with the extracts of concentrations 6.3, 12.5, 25, and 50 *µ*g/ml at 37°C in a 5% carbon dioxide incubator. The negative control contained cells and RPMI complete media only, and positive control contained cells. The results are mean ± SD using Dunnett's multiple range test (*p* < 0.05) compared to control. ^*∗*^*p* < 0.05; ^*∗*^^*∗*^*p* < 0.01; ^*∗*^^*∗*^^*∗*^^*∗*^*p* < 0.001.

The extracts and phytochemical-enriched fractions obtained from *P. curatellifolia* leaves were tested for cytotoxic activity against immune cells obtained from mice peritoneal cavity. This assessment measures cell death, inhibition of cell growth, or cell proliferation. Reduction of cell viability by more than 30% is considered a cytotoxic effect [25]. A typical MTT assay plate is shown in Figure 6. All *P. curatellifolia* extracts had no significant cytotoxic activity (*p* > 0.05) when compared to results for cells only. Of note, was the water extract which exhibited significant proliferative activity (*p* < 0.001) of the murine immune cells at all extract concentrations (Figure 7).

## 4. Discussion

Toxicity testing can reveal some of the risks that may be associated with the use of medicinal plants especially in sensitive populations [25]. Several related plants from the genus *Parinari* have been assessed for toxicological activity in both *in vitro* and *in vivo* model systems. These plants are *P. exelsa*, *P. capensis*, *P. congenis*, *P. polyandra*, *P. campestris*, and *N. macropylla. N. macrophylla* is fairly toxic [26], and phytochemicals obtained from *P. capensis* have been found to possess potent antifungal and antimalarial effects [27, 28]. *P exelsa*, *P. polyandra*, *P. congenis*, and *P. campestris* are nontoxic [29–32]. Generally, most species in the genus are nontoxic and this observation aids in predicting the safety profile of *P. curatellifolia* leaves. Toxicological screening using some of the most important body cells such as red blood cells and immune cells is essential in determining the plant's safety profile.

The most abundant cells in the human body are the erythrocytes, which own many biological and morphological characteristics; hence, they have been widely exploited in drug transport and biocompatibility studies [33]. The erythrocyte model has been widely used as it presents a direct indication of toxicity of injectable formulations as well as a general indication of membrane toxicity. Another advantage of erythrocytes model is that blood is readily available and that cells are easy to isolate from the blood; moreover, its membrane has similarities with other cell membranes [34]. Many plants contain chemical substances that might have a haemolytic or antihaemolytic effect on erythrocytes. Studies have indicated that the membranes of human erythrocytes from blood types have varying stability as determined from the mean corpuscular fragility. Plant extracts can positively affect the red cell membrane and many plants have serious adverse effects, which include induction of haemolytic anaemia [4]. The ability of the plant extracts to lyse red blood cells at varying degrees could be attributed to the presence of different types of saponins where different saponins show different levels of haemolytic activity [34]. *P. curatellifolia* water extract had the highest haemolytic activity among all solvent extracts, and the saponin fraction had the highest haemolytic activity among all the extracts and phytochemical-enriched fractions tested.

Water is the most polar solvent compared to all the other solvents used, and its extraction spectrum contains the most polar biomolecules such as carbohydrates and other saponins that are not well extracted by other polar solvents such as methanol and ethanol [35]. Saponins are a structurally diverse family of plant secondary metabolites that possess the ability to haemolyse erythrocytes. Since saponins are polar compounds, the extraction methods use water, aqueous methanol or ethanol, absolute ethanol, methanol, or *n*-butanol [36]. The type of sugar moiety and functional group attached to a sugar moiety and the concentration of saponin influence the ability of the saponin to dissolve in different solvents, and also different extracts exert different activities [37].

Haemolysis of erythrocytes appears to result from saponin ability to form complexes with cell membrane cholesterol, resulting in pore formation, increased cell permeability, and alterations in the negatively charged carbohydrate portions on the cell surface [38]. Molecular dynamic simulation technique was used to demonstrate the haemolytic mechanism of saponins [39, 40]. Saponins initially penetrate easily into the lipid bilayer and accumulate in the lipid raft microdomain. Lipid rafts are microspheres within cell membranes that are enriched in cholesterol and sphingolipids. Saponins then bind to cholesterol in the lipid membrane and block cholesterol from interacting with sphingomyelin. Binding of saponins to cholesterol affects the delicate effect of sterols as membrane stabilizers. The saponin-cholesterol micelles destabilise the structure of the lipid raft and disrupt the lipid bilayer, which eventually leads to the haemolysis of the erythrocytes [37]. Therefore, the higher the concentrations of saponins within the extract, the higher the haemolytic activity observed.

Cytotoxicity is generally used to predict potential toxicity using cultured cells that may be normal or transformed cells such as mouse peritoneal cells [41]. The test typically involves short-term exposure of cultured cells to test compounds to identify how the compound may affect basal or specialised cell functions, before performing toxicological studies in whole organisms. It can also provide an understanding of the carcinogenic and genotoxic nature of herb-derived substances and extracts. The capacity of a plant extract to inhibit cellular growth and viability can also be determined as an indication of its toxicity [42].

Assessment parameters include inhibition of cell proliferation, cell viability indicators, and morphologic and intracellular differentiation markers [43]. In conducting cytotoxicity, it is important to consider factors such as cell culture systems and methods which affect test outcomes. For instance, some cell types may not be compatible with the solvent used to prepare the test solutions. Many plant extracts and compounds are nonpolar and prepared as solutions in dimethyl sulfoxide before cytotoxicity testing. DMSO has been reported to be cytotoxic at certain concentrations, and this effect varies between cell types. Therefore, it is often necessary to predetermine the maximum tolerable solvent concentration in cytotoxicity assays especially during validatory stages, and control using the carrier solvent alone must be used in the cytotoxicity testing [44]. In this assay, the DMSO concentration that did not interfere with the assay was 0.1%.

*P. curatellifolia* extracts did not show cytotoxicity towards the mouse lymphocytes. The water extract showed significant (*p* < 0.001) proliferative potential towards the peritoneal cells, and this could be attributed to the presence of several biomolecules within the water extract. The water extract is known to extract compounds such as anthocyanins, starch, polypeptides, saponins, terpenoids, and lectins, which increase T-cell and B-cell proliferation [45–47]. Anthocyanins have been found to protect the thymus and spleen and increased lymphocyte proliferation, suggesting that there is improved immunity [48]. Most terpenoids have been shown to increase proliferation of mouse spleen cells and B lymphocytes, while some terpenoids only stimulate the proliferation of B cells but inhibit T-cell proliferation [49]. Plant polypeptides have been shown to promote the proliferation of macrophages, TNF-*α* and IL-6 secretion, TLR2 and TLR4 expression, and increase macrophage phagocytic ability through NO and hydrogen peroxide release [50].

Although most saponins have a strong binding affinity for cholesterol, only some stimulate the immune system [51]. This could be a probable reason why saponin-enriched fraction did not have any proliferative properties towards the mouse immune cells. Saponin-based adjuvants are used in animal and human cancer vaccines, as they induce protective cellular immunity. For example, the saponin QS-21 isolated from *Quillaja saponaria* stimulate both antibody-based humoral immune responses (Th_2_) and cellular immunity (Th_1_), including the production of antigen-specific cytotoxic T-lymphocytes [52].

Lectins, a group of phytochemicals extracted with water, also stimulate the proliferation of T cells and B cells. Lectins are carbohydrate-binding proteins that are found in plants. Lectins such as Con-A, originally isolated from jack bean (*Canavalia ensiformis*), have been found to possess the powerful mutagenic activity and are known to induce mouse T cells but not B cells despite similar binding affinities to both [53]. The presence of lectins at the start of cell culture initiation is thought to help in stimulating maximal lymphocyte response. The water extract, with a cocktail of immunostimulatory phytochemicals, can potentially be used to develop phytochemicals that may be used in cancer therapy or as adjuncts to therapy.

## 5. Conclusion

*P. curatellifolia* leaf extracts did not exhibit any toxic effects towards both erythrocytes and immune cells in this model of study. The water extract was shown to stimulate the immune system by increasing the proliferation of T cells and B cells. These results show a lack of toxicity on mammalian cells and, therefore, support the use of *P. curatellifolia* leaf extracts in the treatment of various infections in folk medicine.

## Figures and Tables

**Table 1:** Physical properties and percentage yield of three phytochemical-enriched fractions.

Enriched fraction	Colour and consistency after drying	Percentage yield
Alkaloid	Brown, solid	1.26
Flavonoid	Dark brown, solid	4.59
Saponin	Golden brown, solid	1.81

## Data Availability

The datasets used and/or analysed during the current study are available from the corresponding author on reasonable request.
